# Using Low-Cost Sensors to Assess Fine Particulate Matter Infiltration (PM_2.5_) during a Wildfire Smoke Episode at a Large Inpatient Healthcare Facility

**DOI:** 10.3390/ijerph18189811

**Published:** 2021-09-17

**Authors:** Phuong D. M. Nguyen, Nika Martinussen, Gary Mallach, Ghazal Ebrahimi, Kori Jones, Naomi Zimmerman, Sarah B. Henderson

**Affiliations:** 1 Environmental Health Services, BC Center for Disease Control, Vancouver, BC V5Z 4R4, Canada; phuong.nguyen@bccdc.ca; 2 Department of Mechanical Engineering, The University of British Columbia, Vancouver, BC V6T 1Z4, Canada; nmartinu@student.ubc.ca (N.M.); nzimmerman@mech.ubc.ca (N.Z.); 3 Air Health Sciences Division, Health Canada, Ottawa, ON K1A 0K9, Canada; gary.mallach@canada.ca; 4 Provincial Health Services Authority, Vancouver, BC V6H 4C1, Canada; ghazal.ebrahimi@phsa.ca; 5 Vancouver Coastal Health, Vancouver, BC V5Z 1A1, Canada; kori.jones@vch.ca; 6 School of Population and Public Health, The University of British Columbia, Vancouver, BC V6T 1Z3, Canada

**Keywords:** indoor air quality, PM_2.5_, wildfire smoke, infiltration, low-cost sensors, healthcare facility

## Abstract

Wildfire smoke exposure is associated with a range of acute health outcomes, which can be more severe in individuals with underlying health conditions. Currently, there is limited information on the susceptibility of healthcare facilities to smoke infiltration. As part of a larger study to address this gap, a rehabilitation facility in Vancouver, Canada was outfitted with one outdoor and seven indoor low-cost fine particulate matter (PM_2.5_) sensors in Air Quality Eggs (EGG) during the summer of 2020. Raw measurements were calibrated using temperature, relative humidity, and dew point derived from the EGG data. The infiltration coefficient was quantified using a distributed lag model. Indoor concentrations during the smoke episode were elevated throughout the building, though non-uniformly. After censoring indoor-only peaks, the average infiltration coefficient (range) during typical days was 0.32 (0.22–0.39), compared with 0.37 (0.31–0.47) during the smoke episode, a 19% increase on average. Indoor PM_2.5_ concentrations quickly reflected outdoor conditions during and after the smoke episode. It is unclear whether these results will be generalizable to other years due to COVID-related changes to building operations, but some of the safety protocols may offer valuable lessons for future wildfire seasons. For example, points of building entry and exit were reduced from eight to two during the pandemic, which likely helped to protect the building from wildfire smoke infiltration. Overall, these results demonstrate the utility of indoor low-cost sensors in understanding the impacts of extreme smoke events on facilities where highly susceptible individuals are present. Furthermore, they highlight the need to employ interventions that enhance indoor air quality in such facilities during smoke events.

## 1. Introduction

Wildfire smoke may become the dominant source of exposure to fine particulate matter (PM_2.5_) in western North America, which has uncertain human health implications [1]. Short-term exposure to wildfire smoke is associated with an increased risk of acute respiratory outcomes, such as exacerbations of asthma and chronic obstructive pulmonary disease (COPD) [2,3,4,5,6]. Within Canada, short-term exposures during the 2013–2015 and 2017–2018 wildfire seasons were associated with 54–250 deaths due to acute cardiorespiratory outcomes, while long-term exposures were associated with 570–2500 deaths due to chronic disease [7]. Although such estimates are typically made using PM_2.5_ ambient air quality data, these outdoor concentrations do not explicitly assess variability in indoor PM_2.5_ exposure. Most people in North America spend at least 85% of their time inside a home or other building [8], meaning that most health effects associated with ambient smoke are due to exposures that occur indoors. As wildfire seasons are expected become longer and more intense [9], it is critical that we study smoke infiltration and effective interventions, particularly in settings that house susceptible populations [10].

The indoor infiltration of outdoor wildfire smoke has not been comprehensively studied, so it remains unclear how much protection remaining indoors provides during wildfire smoke episodes. In addition, there are limited data for multi-story non-residential buildings such as offices and healthcare facilities. Healthcare facilities are particularly concerning because they serve individuals who are more susceptible to smoke exposure due to compromised health status [3,11]. One study during the 2018 Camp Fire may provide some insight. Pantelic et al. (2019) compared two large commercial buildings, one with a mechanical ventilation system (HVAC) that was outfitted with two-staged particle filtration, and one without. The building with HVAC had a mean indoor PM_2.5_ concentration of 21 µg/m^3^ and an indoor-to-outdoor ratio of 0.27. In comparison, the building with natural ventilation had a mean indoor concentration of 36 µg/m^3^ and an indoor-to-outdoor ratio of 0.67 [12]. The latter is more consistent with infiltration coefficients typically reported for single-family residences, which rarely have outdoor air intakes to filter incoming air, and rely on natural ventilation and leaks in the building envelope for air exchange. For example, the mean infiltration factor for 17 homes in southern British Columbia, Canada during the 2004–2005 wildfire seasons was 0.61 [13].

Differences between buildings suggest that airtightness, filtration and ventilation play important roles in outdoor PM_2.5_ infiltration during wildfire smoke episodes [12]. A more airtight envelope reduces smoke penetration through cracks and crevices, and mechanical ventilation typically provides at least some filtration of incoming outdoor air. Furthermore, it may be possible to increase filtration of fresh or recirculating air by upgrading and/or reducing air bypass around filters. It is also possible to reduce the fresh air intake on some HVAC systems during smoke episodes, though this is antithetical to best practice for COVID-19 risk reduction [14]. Even when measures are taken to reduce smoke infiltration, indoor PM_2.5_ concentrations vary widely between different buildings and residential homes during smoky periods [15,16]. Methods that evaluate infiltration on a building-by-building basis may be needed to inform actions taken by facility operators and HVAC specialists, especially for facilities where susceptible individuals reside. Low-cost sensors may have an important role to play in evaluating and optimizing building-specific interventions in both existing buildings and new construction projects. Notably, the building stock for hospitals, rehabilitation centers, long-term care, residential care, and publicly-funded housing is highly variable, with many older facilities constructed under outdated building codes. There is currently no comprehensive information about wildfire smoke infiltration in such environments.

Another factor underlying infiltration is meteorological conditions, such as precipitation, wind speed, wind direction, temperature, and relative humidity. Some studies have reported that wind speed, temperature, and ambient relative humidity are negatively correlated with PM_2.5_ concentrations and the PM_2.5_ indoor–outdoor ratio [17,18,19,20]. Precipitation is also inversely proportional to PM_2.5_ concentrations, due to the removal effect where particulate matter is removed from the air to the surface by rain [17]. However, there are some studies that show no correlation or negligible effects of temperature and precipitation [18,21,22], while others show positive relationship between temperature and PM exposure, due to increased window opening for ventilation and cooling [23].

There are many factors affecting infiltration of wildfire smoke, and there is a real opportunity to use low-cost sensors to better understand the impacts on specific buildings. One caveat is that the data quality from low-costs sensors can be uncertain because they are sensitive to many variables, including relative humidity, temperature, particle morphology, and particle composition [24]. Correcting for these effects is necessary to yield higher-quality data for analysis. When evaluated under outdoor field conditions, low-cost PM_2.5_ sensors are generally well-correlated with reference instruments, but they tend to over-predict concentrations in both ambient and wildfire smoke conditions [25]. Nonetheless, linear correction has been effective for reducing errors [25,26]. Although their performance indoors has not been widely evaluated, their outdoor performance suggest that indoor networks of low-cost sensors can significantly improve our understanding of indoor air quality and its health impacts, especially during significant outdoor air quality events [27].

British Columbia experienced extreme wildfire seasons in 2017 and 2018, including episodes of prolonged smoke that affected multiple healthcare facilities. In response, local health authorities began planning to outfit three inpatient healthcare facilities with multiple indoor and outdoor low-cost PM_2.5_ sensors. This initiative was intended to support and inform future planning, design, and operational measures required to reduce the health risks associated with outdoor air quality for patients and staff in existing and future facilities. Sensors were installed at a rehabilitation facility in Vancouver in August 2020, with the intention of leaving them in place for at least one year. About 2 weeks later, dense smoke was transported into southern British Columbia from wildfires in California, Oregon, and Washington states. As such, we have a unique opportunity to examine the impacts of wildfire smoke on an inpatient rehabilitation facility.

In this study, we describe methods used to calibrate the low-cost sensor data using measurements from the surrounding regulatory network. After data calibration, we summarize indoor and outdoor PM_2.5_ concentrations at the healthcare facility during the smoke episode and during typical days on either side of the smoke episode. Specifically, we remove indoor peaks from the indoor measurements, calculate the coefficient of infiltration at each indoor location, and examine the speed of infiltration into the facility.

## 2. Materials and Methods

### 2.1. Study Context and Period

Greater Vancouver is a major urban center on the southwestern coast of British Columba, Canada. In 2020, its population was approximately 2.5 million people. The region typically has excellent ambient air quality, with an annual average PM_2.5_ concentration of approximately 5.0 µg/m^3^. Days exceeding the 24-h objective of 25 μg/m^3^ are rare, except during wildfire seasons when the region can experience markedly higher concentrations of PM_2.5_ originating from fires in British Columbia and other parts of western North America. Our analyses cover the period from August 21 through 31 October 2020. For all analyses we defined the *smoke episode* as September 8 through 18, which corresponds with the timing of the 11-day air quality advisory issued by Metro Vancouver. We defined the *typical days* as August 21 through September 7 and September 19 through October 31.

### 2.2. The Rehabilitation Facility

The facility is a large multi-story rehabilitation center that usually serves inpatient and outpatient populations, but all outpatient services were suspended during the study period due to the COVID-19 pandemic. The facility provides services in four different programs: (1) acquired brain injury; (2) spinal cord injury; (3) arthritis; and (4) neuromusculoskeletal. The main building was constructed in 1972. It has four floors above ground and a basement, and most rehabilitation activities occur on the ground floor in the physical and occupational therapy rooms. However, most therapy programs were offered in patient rooms during the study period in accordance with the pandemic safety plan. Offices and patient rooms are located on the second to fourth floors, and the basement serves a range of operational functions, including the loading bay.

Heating for the facility is provided by radiators, and cooling is provided by an HVAC system with ten air handling units. Most of the windows are fully operable. During the smoke episode the pre-filters in the air handling units were changed more frequently, the cooling system was operational, and building occupants were asked to keep the windows closed. No other special precautions were taken to limit indoor smoke impacts. Due to COVID-19 protocols, entrance to the building was only possible through two doors: one in the main lobby for patients and visitors, and one at the side of the ground floor for staff.

### 2.3. Low-Cost Sensors

The 2018 model of the Air Quality Egg (EGG) by Wicked Device was used for this study (version 2.0) [28]. All the EGGs had dual Plantower PMS5003 (2016) sensors that measure PM_2.5_ concentrations via light scattering, with the reported value being the average of the two sensors. The Plantower sensors report an effective range between 0–500 µg/m^3^ and a maximum consistency error of ±10% at 100–500 µg/m^3^ and ±10 µg/m^3^ at 0–100 µg/m^3^. Each EGG also had a temperature and relative humidity sensor. Some EGGs used in the study also had sensors for carbon dioxide (CO_2_), nitrogen dioxide (NO_2_), and volatile organic compounds (VOC), but these data are not included in the analyses. The EGGs log measurements from all sensors at 1-min intervals and automatically upload the data to a central repository.

### 2.4. Sensor Deployment at the Facility

**Figure 1:**
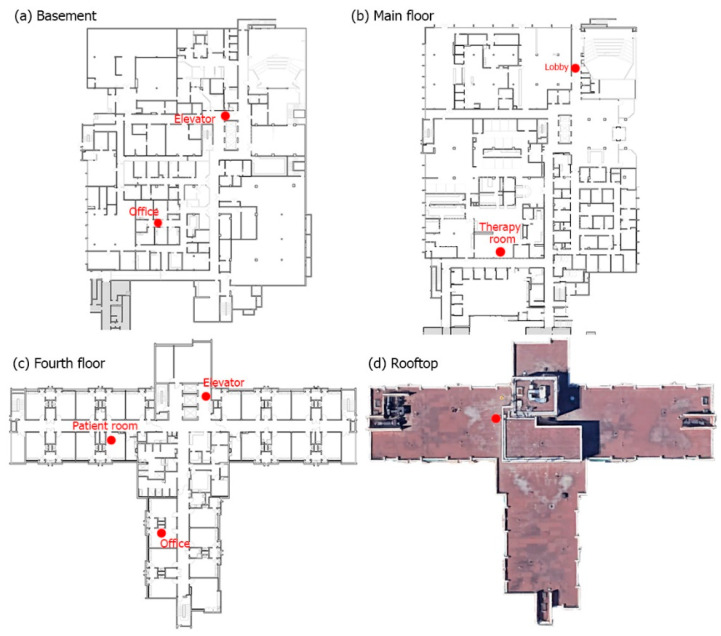
Floor plan of the facility with locations of low-cost sensors (red dots).

Nine EGGs were deployed to the facility in August 2020, with two placed outdoors and seven placed indoors at a height of approximately two meters. One outdoor EGG was placed on the roof of the main building, and one was placed outside of a window on the second floor. The latter EGG failed early in the study period and was excluded from the analyses. Three indoor EGGs were placed near the elevator doors in the basement, in the ground floor lobby, and on the fourth floor. The remaining EGGs were placed in a basement office, the ground floor therapy room, a fourth-floor office, and a fourth-floor patient room (Figure 1). The placement of the nine sensors was discussed with subject matter experts to identify locations that would provide useful information for the study objectives.

### 2.5. Collocation of the Low-Cost Sensors with Federal Equivalency Methods (FEM)

**Figure 2:**
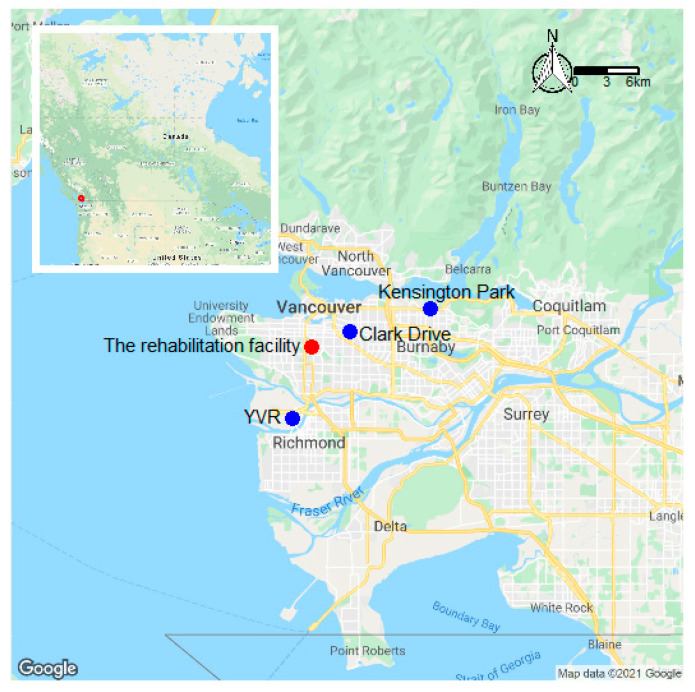
Locations of the rehabilitation facility (red dot) and three National Air Pollution Surveillance (NAPS) monitoring stations (blue dots) at Vancouver International Airport (YVR), Clark Drive, and Kensington Park in greater Vancouver, Canada. Location of greater Vancouver relative to North America (red square on inset map).

Six EGGs from the larger project were co-located at the Kensington Park ambient air quality monitoring station (Figure 2) from 17 July through 30 July 2020. This station is part of the National Air Pollution Surveillance (NAPS) network that measures PM_2.5_ using beta attenuation monitors that meet Federal Equivalency Method (FEM) standards. Two of the co-located EGGs (EGG 4 and EGG 6) were later deployed to the facility, in the ground floor lobby and outside on the second floor, respectively. Again, EGG 6 failed early in the study period and was excluded from the facility analyses.

### 2.6. Data Calibration with a Regional Baseline Model

The average PM_2.5_ concentrations at Kensington Park in July 2020 were low and did not represent concentrations during the smoke episode, so we developed and applied a novel approach using data from FEM monitors located at the Vancouver International Airport (YVR) and Clark Drive NAPS stations during the study period. These stations are located to the southwest and northeast of the facility, respectively (Figure 2). To check the accuracy of the calibration model it was applied to the six EGGs that were co-located with the FEM monitor at Kensington Park.

All data management, analyses, and visualization for this study were conducted using R version 4.0.3 [29]. Measurements from the EGGs and NAPS stations were available at 1-min resolution, which were smoothed to 15-min intervals using the *timeAverage* function of the *openair* package [30]. The following measurements were collected from each EGG for this study: Temperature (T, °C); relative humidity (RH, %); and PM_2.5_ (µg/m^3^).

The EGG PM_2.5_ data collected at the facility rooftop were used to train a calibration model with the average baseline of the FEM PM_2.5_ concentrations (i.e., estimated regional PM_2.5_ concentration) in a multiple linear regression that also included temperature, relative humidity, and dewpoint parameters, as suggested elsewhere [31]. The EGG dew point was computed by the Magnus formula [32] as shown in Equation (1):

(1) $$DP\left(T,\;RH\right)=\frac{\lambda \;\cdot (\ln\left(\frac{RH}{100}\right)+\frac{\beta \;\cdot T}{\lambda +T})}{\beta -(\ln\left(\frac{RH}{100}\right)+\frac{\beta \;\cdot T}{\lambda +T})}\;$$

where λ = 243.12 °C; β = 17.62; T = temperature measured by the EGG; and RH = relative humidity measured by the EGG.

The data for the calibration model were taken from a 5-week period, from August 21 at 08:00 until September 24 at 08:00. This period was chosen because the rooftop EGG data were relatively continuous throughout. A Kalman smoothing function was applied to the rooftop EGG PM_2.5_ and FEM PM_2.5_ data to impute any missing values. The baseline for each time series was then computed using a Rolling Ball algorithm [33], and the average of the FEM baselines was taken. The baselines were computed using the *baseline* package in R [33]. Assuming the average of the FEM PM_2.5_ concentrations to be the true value, EGG measurements of PM_2.5_, temperature (T), relative humidity (RH) and dewpoint (DP) were used to predict the FEM baseline and the resulting equation (Equation (2)) was applied to all EGG PM_2.5_ data collected at the facility.

Corrected PM_2.5_ = −40.562 + 1.019*PM_2.5_ − 1.7102*T − 0.534*RH + 2.153*DP (2)

To test the accuracy of the model, PM_2.5_ baselines for the FEM collocation period were computed in the same manner as described for the facility datasets. The regional baseline model was then applied to the collocation EGG data and compared with the FEM PM_2.5_ concentrations. Metrics to quantitatively assess the performance of the calibration model included Pearson r, the mean absolute error (MAE) and the coefficient of variation of the mean absolute error (CvMAE), as calculated by Equation (3). These metrics are used to quantify the correlation between the estimated and true concentration of PM_2.5_ (Pearson r), the average of differences between the estimated and true concentration of PM_2.5_ (MAE), and the mean absolute error normalized to PM_2.5_ concentration (CvMAE). A higher Pearson r signifies a strong linear relationship, and lower MAE and CvMAE signify closer agreement between the calibrated and true values of PM_2.5_.

(3) $$CvMAE=\frac{1}{avg.FEM\left[PM_{2.5}\right]}\times \left(\frac{1}{n}\overset{n}{\underset{i=1}{\sum }}\left|calibrated{\left[PM_{2.5}\right]}_{i}-FEM{\left[PM_{2.5}\right]}_{i}\;\right|\right)$$

where *n* = the number of observations in the collocation dataset.

### 2.7. Removal of Indoor-Generated PM_2.5_

Before calculating the infiltration coefficients, we used an algorithm to remove indoor peaks that could have resulted from indoor sources, such as kitchen and manufacturing activities in the facility. The algorithm we used was adapted from a previous paper on infiltration of outdoor PM_2.5_ using 1-h averages [34]. To adapt the approach for 1-min measurements, we modified the algorithm and implemented stricter thresholds to identify indoor peaks. Each indoor peak comprises rising concentrations and decaying concentrations, which were identified as described below.

First, for every time point (t), we checked whether (1) the indoor–outdoor ratio was greater than 2.0, (2) the indoor concentration was at least 2 µg/m^3^, and (3) the indoor concentration had increased from the previous time point (Equation (4)). Outdoor concentrations were taken from the rooftop sensor at the facility. When these conditions were met, the beginning of an indoor peak was identified, and the data point was removed. Second, we checked all measurements following the beginning of an indoor peak and removed them if the concentration continued to rise (Equation (5)). Finally, we removed the decaying side by checking whether (1) the previous measurement had been removed, (2) the indoor–outdoor ratio was greater than 1.0, and (3) the indoor concentration had decreased from the previous time point (Equation (6)).

(4) $$\frac{I_{t}}{O_{t}}>2\;\mathrm{and}\;I_{t}\geq 2\mu g/m^{3}\;\mathrm{and}\;\frac{I_{t}}{I_{t-1}}>1$$

(5) $$I_{t-1}\;\mathrm{was}\;\mathrm{previously}\;\mathrm{removed}\;\mathrm{and}\;\frac{I_{t}}{I_{t-1}}>1$$

(6) $$I_{t-1}\;\mathrm{was}\;\mathrm{previously}\;\mathrm{removed}\;\mathrm{and}\;\frac{I_{t}}{O_{t}}>1\;\mathrm{and}\;\frac{I_{t}}{I_{t-1}}\;\leq \;1$$

where: *I* = the indoor concentrations at time intervals t and *t* − 1; *O* = the outdoor concentration at time interval *t*.

### 2.8. Smoke Infiltration

The infiltration coefficient represents the proportion of outdoor PM_2.5_ that has infiltrated and persisted indoors. Once the indoor peaks were removed, the infiltration coefficient was quantified using distributed lag linear models and the *dlnm* package [35,36]. In this model, the indoor PM_2.5_ concentration at time (*t*) is estimated by the cumulative lagged effects of the outdoor rooftop PM_2.5_ concentration over a 60-min interval (Equation (7)).

(7) $$I_{t}=\beta_{1}O_{t-1}+\beta_{2}O_{t-2}+\cdots +\beta_{m}O_{t-m}+\;\alpha \;m=1,\;2,\;\ldots \;,60$$

where: $I_{t}$ = the indoor PM_2.5_ concentration at time *t*; $O_{t-m}$ = the outdoor concentration at time t-m minutes; $\beta_{m}$ = the lagged coefficient of $O_{t-m}$; α = the intercept.

Each coefficient represents the contribution and persistence of the outdoor concentration at every previous minute over a 60-min interval on the current indoor PM_2.5_ concentration. The infiltration coefficient (*F_inf_*) is quantified by the sum of the lagged coefficients (Equation (8)). We used the 1-min calibrated indoor EGG PM_2.5_ with peaks removed and rooftop EGG PM_2.5_ for the calculations during periods that capture the smoke episode and typical days. Missing values were omitted.

(8) $$F_{inf}=\beta_{1}+\beta_{2}+\ldots +\beta_{m}\;m=1,2,\ldots ,60$$

To qualitatively examine the persistence of indoor smoke, we used stacked heat maps of EGG PM_2.5_ concentrations on the facility rooftop and indoors. Each plot shows the smoke period compared with the pre- and post-smoke periods, by hours of the day.

### 2.9. Meteorological Conditions

The EGGs measure temperature and relative humidity, so we were able to examine differences in these parameters at the facility during the smoke episode and the typical days. Although we could not measure complete meteorology at the facility, we were able to access information on temperature, humidity, wind speed, wind direction, and precipitation at the FEM sites at YVR, Clark Drive, and Kensington Park. We compared meteorological parameters during the smoke episode and the typical days.

## 3. Results and Discussion

### 3.1. Calibration Model Performance

Prior to any analyses with the EGG data collected at the facility, they were calibrated using the regional baseline model described above (Equation (2)). The magnitudes and directions of the model coefficients were comparable with those previously reported for similar models [31]. When tested on the collocation period data, Pearson r values ranged from 0.73–0.83 among the EGGs, MAE ranged from 2.61–4.88 µg/m^3^, and CvMAE ranged from 0.48–0.91 (Table 1). The aggregate performance of the calibration models was relatively strong, with mean Pearson r, CvMAE, and MAE values of 0.80, 0.58, and 3.10, respectively. The average concentration at the Kensington Park ambient air quality monitoring station during the collocation period was very low (4.47 µg/m^3^), which accounts for the high CvMAE values during our calibration model assessment. The EGGs also reported very low average PM_2.5_ levels during this period (Table 1).

The 24-h average of uncalibrated PM_2.5_ concentrations during the typical days and smoke episode were 8.7 µg/m^3^ and 73.1 µg/m^3^, respectively In comparison, the 24-h average of calibrated PM_2.5_ concentrations were 7.5 µg/m^3^ and 72.0 µg/m^3^, respectively. The preliminary results of our regional baseline regression have implications for future low-cost sensor calibration studies. When collocation data are not available or representative of the outdoor conditions at the time the study is performed, we suggest that a calibration model can be developed from a regional PM_2.5_ baseline that reflects the surrounding region during the study period. Use of regional baseline calibration may drastically reduce the need for lengthy collocation periods when performing measurements of pollutants from outdoor sources with low-cost sensors, especially for citizen science projects that require large and distributed sensor networks. We recommend this as an area for future study, specifically as it applies to extreme air quality events such as wildfire smoke episodes.

### 3.2. Indoor Peak Removal

We applied an algorithm that removed peaks caused by indoor PM_2.5_ sources at the facility, and the number of peaks varied by location (Table 2). The ground floor therapy room and a patient room on the fourth floor had the most indoor peaks. The therapy room had known indoor sources such as a model kitchen, a facility for manufacturing assistance aids, and supplies for hot wax therapy, all of which were used by staff during working hours. Indoor sources of PM_2.5_ in the patient room are unknown.

### 3.3. Indoor and Outdoor PM_2.5_ Concentrations

**Figure 3:**
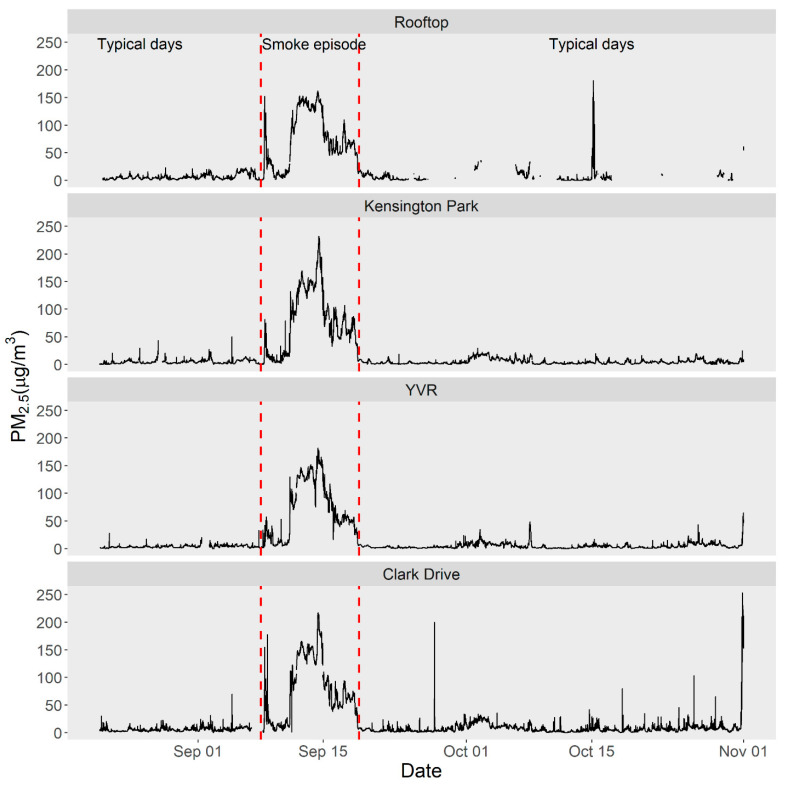
Time series of raw 1-min PM_2.5_ concentrations from the low-cost sensors located outdoors on the rehabilitation facility rooftop and from beta attenuation monitors at three national air pollution surveillance (NAPS) monitoring stations at Kensington Park, Vancouver International Airport (YVR) and Clark Drive in greater Vancouver, Canada (Figure 2).

**Figure 4:**
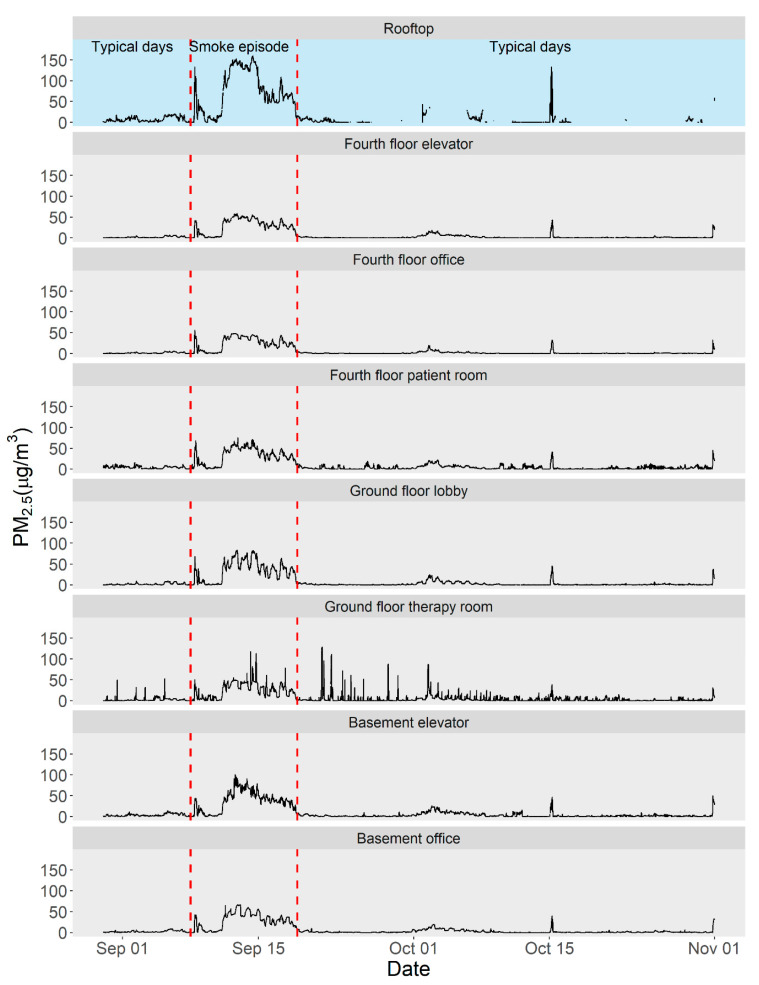
Time series of calibrated 1-min PM_2.5_ concentrations from the low-cost sensors located outdoors (blue) and indoors (grey). Indoor peaks have not been removed from these data. The cause of the short peak on October 15 is unknown.

Air quality in greater Vancouver was excellent on the typical days during the study period. The 24-h FEM PM_2.5_ averages (range) at Kensington Park, Clark Drive, and YVR were 4.8 (1.0–17.6), 7.1 (1.5–46.2), and 4.5 (0.9–14.3) µg/m^3^, respectively (Figure 3). Higher concentrations at Clark Drive reflect its roadside location on a heavily trafficked trucking route. In comparison, the calibrated 24-h PM_2.5_ average at the facility rooftop was 7.5 (0.0–46.8) µg/m^3^ (Figure 4). During the wildfire smoke episode, the rooftop EGG at the facility had a 24-h average of 72.0 (7.7–141.6) µg/m^3^. In comparison, the FEM monitors at Kensington Park, Clark Drive, and YVR had 24-h averages of 76.1 (7.7–172.4), 75.8 (11.1–161.4), and 69.1 (9.5–144.9) µg/m^3^, respectively, during the smoke episode.

**Figure 5:**
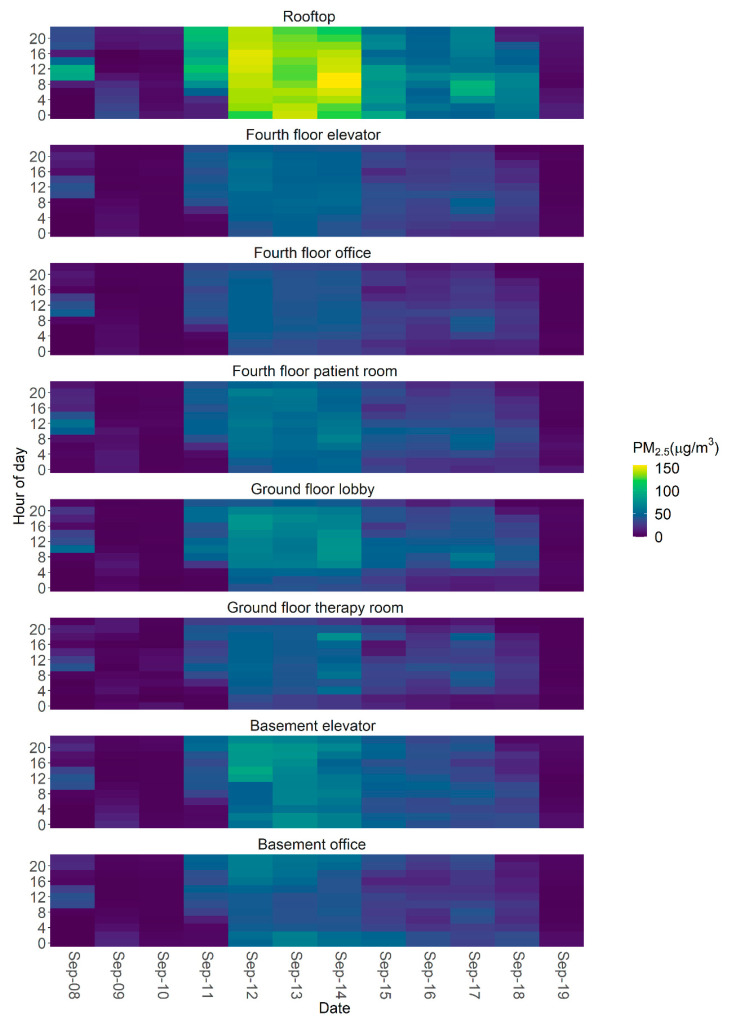
Heat maps of 2-h averaged PM_2.5_ concentrations at the rehabilitation facility during the wildfire smoke episode. The low cost sensor on the rooftop is shown at the top, and those located indoors are shown below. The y-axis of each plot indicates the time of day. Indoor peaks have not been removed from these data. Data from the morning of September 11 show that smoke moved rapidly indoors as the rooftop concentration started to increase, and overnight data from September 18–19 show that smoke cleared rapidly out of the building as rooftop concentration decreased.

On average, the indoor PM_2.5_ concentrations at the facility were substantially higher during the wildfire smoke episode than on the typical days, with an average 24-h value of 29.6 µg/m^3^ compared with 2.4 µg/m^3^, respectively. The indoor PM_2.5_ concentrations varied by location, with higher concentrations observed near entrances and exits (Table 3 and Figure 5). During both the smoke episode and the typical days, indoor PM_2.5_ concentrations were, on average, lower than the outdoor concentration on the rooftop, which is consistent with other studies conducted in large buildings [12,37].

### 3.4. Indoor Infiltration of Outdoor PM_2.5_

Infiltration of outdoor PM_2.5_ was generally higher during the wildfire smoke episode than on typical days, but it varied by location (Table 2). On typical days, the mean (range) infiltration was 0.32 (0.22–0.39) across all indoor locations, and infiltration was highest at the basement elevator and lowest in the ground floor therapy room (Table 2). During the smoke episode, the mean infiltration increased to 0.37 (0.31–0.47), an average increase of 19%, ranging from a decrease of 3% in the fourth-floor patient to an increase of 41% ground floor therapy room. Although the average infiltration was higher during the smoke episode than on typical days, the patient room on the fourth floor observed no substantial change in infiltration during the smoke episode, after censoring indoor-generated peaks (Table 2). The higher infiltration in the patient room during typical days could be due to occupants opening the windows.

Overall, the coefficients suggest that there is more infiltration of PM_2.5_ when the outdoor concentrations are very high. However, the mean infiltration into the facility during the wildfire smoke episode was considerably lower than reported mean wildfire smoke infiltration for residential homes, which has ranged from 0.56 to 0.79 in previous studies [13,34,38]. This could suggest that the facility building is more protected against infiltration than private residences, or that management of the building during the COVID-19 pandemic helped to protect it. Even so, indoor concentrations at the facility were high compared with benchmarks such as the air quality objective for Metro Vancouver, which is a 24-h average PM_2.5_ concentration less than 25 µg/m^3^. If the provincial Air Quality Health Index (AHQI) is applied, any 1 h concentration greater than 60 µg/m^3^ is in the high risk category [39], including: 50 h near the basement elevator during the 264 h smoke episode; 48 h in the lobby; 14 h in the fourth floor patient room; 11 h in the basement office; and 3 h in the ground floor therapy room.

The infiltration coefficients calculated for the facility are more consistent with results from previous studies conducted in similar large buildings during an extreme wildfire episode. For example, Pantelic et al. reported a value of 0.27 for a large commercial building with HVAC in Berkeley, California when the median hourly outdoor concentration was 21 µg/m^3^. Similarly, Wheeler et al. reported a value of 0.31 for a public library in Port Macquarie, Australia when the mean outdoor concentration was 30.7 µg/m^3^ [12,37]. Our infiltration coefficients are also consistent with another study that calculated a median infiltration of 0.45 among commercial buildings in Oregon and California during the September 2020 smoke episode [40]. However, the mean outdoor PM_2.5_ concentration was not reported for this study. Guidelines from the Canadian Standards Association (CSA) suggests that occupational therapy and physiotherapy health facilities should maintain 6–9 total air changes per hour (ACH) [41], which might make them more susceptible to smoke infiltration if the incoming air is not effectively filtered. The ACH for the building with HVAC in the Berkley study was below 0.30 [12], making it very airtight in comparison. We were not able to measure ACH for this study.

Infiltration of PM_2.5_ was non-uniform across locations in the facility during both the wildfire smoke episode and typical days. Certain locations, such as the basement elevator and the lobby, were more susceptible to infiltration, resulting in higher coefficients during both periods (Table 2). These differences between locations were not associated with the floor on which the EGGs were located, as there were higher and lower values in the basement, on the main floor, and on the fourth floor. Very few studies have reported on PM_2.5_ infiltration into multi-story buildings, so it is difficult to evaluate whether these findings are typical. Another study with indoor and outdoor pairs of light-scattering sensors placed at different heights on an 8-story building found differences in concentrations by height, but strong correlation between indoor and outdoor values regardless of height [42]. We would have been able to do more similar analyses if the outdoor EGG on the second story had not failed.

We believe higher infiltration values near the basement elevator and in the lobby are best explained by activities occurring in these two locations. The elevators in the basement are near to the loading bay doors and another service door frequently used by cleaners and staff to access the outside of the building. The EGG by the elevators in the lobby was near the main entrance, which includes two sets of double sliding doors and a 12-foot vestibule between. Both sets of doors are often open at the same time to accommodate patients using mobility aids. Other locations, such as the ground floor therapy room, were better protected against infiltration. While the ground floor therapy room is affected by indoor generation of PM_2.5_, it also has a dedicated air handling unit that may help to dilute the air more quickly than in other areas of the building. When indoor locations were ranked by infiltration coefficient during the smoke episode and typical days, the orders were similar (Table 2), possibly because they always have relatively similar rates of airflow. These results suggest that indoor infiltration of outdoor particles varies consistently by location within the building, and that infiltration on typical days can help to assess potential infiltration during smoke episodes, to help prioritize occupied locations for additional measures such as deploying sufficiently sized portable air cleaners. This finding also has implications for space design and functional programming developed for new construction of healthcare facilities.

Infiltration of PM_2.5_ during the smoke episode at the facility may have been mitigated by smoke-specific protocols and restrictions implemented for the COVID-19 pandemic. The pre-filters in the air handling units were changed more frequently during the smoke episode, the cooling system was operational, and building occupants were asked to keep the windows closed. In addition, the COVID-19 safety plan limited building entrances to two doors instead of the usual eight. All patients used the sliding double doors in the ground floor lobby and staff used a different, smaller door at the side of the building. In general, local-level interventions such as portable air cleaners for areas that have higher infiltration might improve conditions without the need for a larger-scale intervention. For example, enforced use of the double door vestibule during smoky conditions could further limit infiltration into the main lobby. Likewise, those without mobility aids could be directed through smaller doors.

### 3.5. Meteorology during Smoke Episode and Typical Days

There was little to no precipitation during both the smoke episode and the typical days (Table 4). Outdoors, the wind speed and relative humidity were, on average, lower during the smoke episode than during the typical days, though the temperatures were somewhat higher. The average wind direction was consistent across both periods and typical for the coastal region. Indoors, both temperature and relative humidity were higher during the smoke episode than during the typical days. These small differences in outdoor and indoor conditions may have had small effects on the infiltration coefficients based on prior literature [17,18,19,20,23].

### 3.6. Indoor PM_2.5_ Patterns

The daily heat map (Figure 5) shows the overall pattern of the smoke episode. We found that the lag between rooftop PM_2.5_ concentration changes and subsequent indoor changes was short, occurring within 1–2 h. When outdoor PM_2.5_ concentrations increased sharply at the facility rooftop on the morning of September 11, indoor PM_2.5_ concentrations across all locations increased within the same time frame, though the impact varied by location. This is consistent with recent reports of rapid outdoor PM_2.5_ infiltration, where approximately half of the total penetration occurred within the first hour [43]. When rooftop PM_2.5_ concentrations decreased, indoor levels also changed quickly to reflect outdoor conditions. Indoor PM_2.5_ concentrations began to decrease quickly after the rooftop PM_2.5_ concentrations began to drop at approximately 18:00 on September 18. This suggests that outdoor PM_2.5_ changes, whether increases or decreases, affected indoor air quality almost immediately, but not uniformly (Figure 5). This highlights the utility of air quality advisories and wildfire smoke forecasting as triggers to implement building-specific smoke readiness plans, as recently recommended by ASHRAE [44].

### 3.7. Limitations

This study has several limitations. First, it was conducted during the COVID-19 pandemic and its associated building restrictions, such as reduced points of entry, suspension of outpatient programs, and no inpatient visitations. These restrictions may have lowered infiltration, and we do not know how smoke would have infiltrated during an extreme episode under normal circumstances. Even so, some of these restrictions may provide insight into protecting large buildings from wildfire smoke infiltration through simple measures such as limiting the number of entrances. Second, the indoor EGGs were not co-located with indoor FEM monitors, and we had to calibrate them with outdoor data, which are not representative of conditions inside the facility. Third, we may not have removed all indoor peaks, and there may be some remaining indoor contribution that is not captured by the algorithm that identified peaks. The relative contribution of indoor sources may have been higher during the typical periods, potentially reducing estimates of infiltration compared with the wildfire smoke episode. During the wildfire smoke episode, both indoor and outdoor concentrations were much higher, and any indoor background concentrations would have made a smaller relative contribution to total indoor PM_2.5_, so the infiltration coefficient may have been attenuated.

## 4. Conclusions

This study is the first to evaluate wildfire smoke infiltration into a healthcare facility, where people with compromised health status reside. We found that infiltration during an extreme wildfire smoke episode at the facility was, on average, 19% higher than infiltration during typical days. Indoor concentrations increased across all locations during a smoke episode, suggesting that no locations were completely protected from smoke, though infiltration was higher in areas near to the limited entrances and exits in use during the COVID-19 pandemic. Restricting entrances and using double doors with vestibules may help minimize overall smoke infiltration. Additionally, the indoor air at the facility quickly reflected outdoor changes measured by the rooftop EGG, whether PM_2.5_ concentrations were increasing or decreasing. We also demonstrated the application of multiple low-cost sensors in evaluating indoor air quality during an extreme wildfire smoke episode. We suggest that long-term use of low-cost sensors can aid facility operators in testing and optimizing actions aimed at protecting occupants from wildfire smoke infiltration. These findings could inform the development of building guidelines by local health authorities for both new construction and renovation of healthcare facilities. As wildfire seasons become longer and more intense, understanding smoke infiltration in healthcare facilities is important to reduce indoor exposure for more susceptible populations.

## Figures and Tables

**Table 1:** The 1-min mean PM_2.5_ concentration, the Pearson *r*, the mean absolute error (MAE), and the coefficient of variation of the mean absolute error (CvMAE) of the six project Air Quality Egg (EGG) lost-cost sensors that were co-located at Kensington Park (Figure 2) prior to the study period. The EGG 4 and EGG 6 sensors were later deployed to the facility inside the ground floor lobby and outside a second-floor window, respectively. However, EGG 6 failed shortly after the beginning of the study period.

	Mean PM_2.5_ (µg/m3)	Pearson r	Mean MAE (µg/m3)	Mean CvMAE
EGG 1	5.92	0.82	2.70	0.50
EGG 2	13.77	0.73	4.88	0.91
EGG 3	5.82	0.83	2.61	0.48
EGG 4	7.21	0.78	3.01	0.56
EGG 5	6.95	0.83	2.66	0.49
EGG 6	6.21	0.82	2.73	0.51

**Table 2:** Infiltration coefficients and their relative ranks for indoor locations in the rehabilitation center after removing indoor peaks during the smoke episode and typical days.

Location	Number of Indoor Peaks Removed	Infiltration Coefficient		Rank	
		Typical Days	Smoke Episode	Typical Days	Smoke Episode
Fourth floor patient room	138	0.38	0.37	2	3
Fourth floor office	3	0.26	0.31	5	7
Fourth floor elevator	6	0.33	0.35	4	4
Ground floor lobby	14	0.36	0.45	3	2
Ground floor therapy room	159	0.22	0.31	7	6
Basement office	8	0.27	0.34	6	5
Basement elevator	64	0.39	0.47	1	1

**Table 3:** The 24-h mean and range of PM_2.5_ concentrations during the smoke episode and typical days. Vancouver International Airport (YVR), Clark Drive, and Kensington Park are ambient air quality monitoring stations in the National Air Pollution Surveillance (NAPS) network (Figure 2). At the rehabilitation facility, the rooftop sensor was outdoors while the other seven were indoors. Completeness of datasets is shown as percentages out of 103,680 expected 1-min data points from 00:00 21 August to 23:59 31 October.

Location	Smoke Episode		Typical Days		Data Completeness (%)
	24-h Mean (µg/m^3^)	Range (µg/m^3^)	24-h Mean (µg/m^3^)	Range (µg/m^3^)	
**Greater Vancouver**					
YVR	69.1	9.5–144.9	4.5	0.9–14.3	96.7
Clark Drive	75.8	11.1–161.4	7.1	1.5–46.2	96.1
Kensington Park	76.1	7.7–172.4	4.8	1.0–17.6	97.2
**The facility**					
Rooftop	72.0	7.7–141.6	7.5	0.0–46.8	62.5
Fourth floor patient room	31.8	2.9–55.1	3.5	0.2–14.2	98.4
Fourth floor office	23.9	1.2–44.6	1.1	0.0–8.6	99.8
Fourth floor elevator	28.3	2.0–50.2	1.6	0.0–11.0	98.6
Ground floor lobby	34.0	1.5–63.4	2.0	0.1–13.5	97.0
Ground floor therapy room	24.5	4.0–45.5	3.4	0.3–19.3	99.1
Basement office	28.4	2.6–52.5	1.9	0.0–11.2	99.8
Basement elevator	36.5	2.6–71.8	3.0	0.0–16.2	99.2

**Table 4:** The 24-h average of meteorological conditions during the smoke episode and typical days. All indoor sensors were aggregated in the indoor facility calculations. Outdoor sensors are the rooftop air quality egg, Clark Drive, Kensington Park, and Vancouver International Airport (YVR) which are part of the National Air Pollution Surveillance (NAPS) network.

Location and Parameter	Smoke Episode	Typical Days
Facility indoors		
Temperature (°C)	22.2	21.9
Relative Humidity (%)	48.3	44.4
Facility rooftop		
Temperature (°C)	17.9	15.8
Relative Humidity (%)	66.0	69.8
Clark Drive		
Temperature (°C)	17.7	11.3
Relative Humidity (%)	77.5	84.4
Wind Speed (km/h)	2.7	4.0
Wind Direction (Degree)	153.3	150.1
Precipitation (mm)	0.0	0.0
Kensington Park		
Temperature (°C)	17.8	11.0
Relative Humidity (%)	76.1	84.9
Wind Speed (km/h)	5.7	7.0
Wind Direction (Degree)	149.6	132.2
Precipitation (mm)	0.0	0.0
YVR		
Temperature (°C)	16.8	11.0
Relative Humidity (%)	80.1	82.5
Wind Speed (km/h)	7.8	10.0
Wind Direction (Degree)	165.4	160.6
Precipitation (mm)	0.0	0.0

## Data Availability

Data are not publicly available. However, most data can be visualized in an interactive Shiny web application: https://ehs-bccdc.shinyapps.io/facility_AQdata/. The “Concentrations” tab shows time series of calibrated data of indoor locations after indoor peaks removal along with the calibrated rooftop and raw data from FEM stations. The “Ratio” tab shows the immediate indoor–outdoor ratio during the study period between all indoor locations and the rooftop, fitted with a smoothing spline.
